# News and Notes

**Published:** 1910-07

**Authors:** 


### NEWS AND NOTES.

---

### NEW INTERNES AT THE GRADY HOSPITAL.

The following graduates of the Atlanta College of Physicians and Surgeons and the Atlanta School of Medicine were selected, as a result of competitive examination, internes for the ensuing year at the Grady Hospital of Atlanta: (1) Dr. D. L. Garrett, of A. C. P. S.; (2) Dr. D. F. Winn, A. S. M.; (3) Dr. A. F. Quillian, A. S. M.; (4) Dr. C. H. Harvey, A. C. P. S.; (5) Dr. G. F. Spearman, A. C. P. S.; (6) Dr. F. G. Jones, A. C. P. S.; (7) Dr. A. B. Jones, A. C. P. S.; (8) W. C. Myers, A. C. M.; (9) Dr. J. L. Tyre, A. C. P. S.; (10) Dr. A. W. Wood, A. C. M.

---

### FIFTH DISTRICT MEETING.

The sixth semi-annual meeting of the Fifth Congressional District Medical Society was held in Grant Park, Atlanta, June 15th. Dr. Hugh M. Lokey, president; Dr. W. D. Travis, vice-president; Dr. W. A. Selman, secretary and treasurer, being acting officers. The committee of arrangements consisted of

Dr. J. Ross Simpson, Dr. E. C. Thrash, and Dr. C. R. Andrews.

The following was the program:

MORNING SESSION: 10:00 A. M. TO 1:00 P. M.

1. Invocation.
2. President's Address.
3. Report of Secretary-Treasurer.

READING OF PAPERS.

4. An Interesting Gunshot Wound of the Abdomen—Dr. Willis Jones, Atlanta.
5. Diagnosis and Treatment of Fractures—Dr. Frank Boland, Atlanta.
6. Suprapubic Cystotomy for Stone, with report of five cases—Dr. W. L. Champion, Atlanta.
7. Report of case of Foreign Body in Bronchial Tube—Dr. F. G. Hodgson, Atlanta.
8. Treatment of Fistulous Tracts and Abscess Cavities—Dr. C. R. Andrews, Atlanta.
9. A Demonstration of X-Rays of Bone Lesions Commonly Seen in Orthropedic Practice—Dr. Michael Hoke, Atlanta.
10. Operations on Gasser's Ganglion for the Cure of Trifacial Neuralgia. Report of two cases—Dr. E. G. Jones, Atlanta.
11. Treatment of Ilio Colitis—Dr. Chas. E. Boynton, Atlanta.
12. Pneumonia in Children—Dr. G. K. Varden, Atlanta.
13. Does Eating Sweets Injure the Teeth Locally?—Dr. S. A. Visanska, Atlanta.
14. Some Facts and Fallacies About Coffee—Dr. G. M. Niles, Atlanta.
15. The Mouth and Teeth During Sickness—Dr. Robin Adair, Atlanta.
16. Recent Ocular Therapeutics—Dr. A. G. Hobbs, Atlanta.

1:00 P. M.—BASKET DINNER.

17. Business Meeting.
18. Something Good—Dr. J. S. Todd, Atlanta.
19. Streptococcuc Mucosus Capsulatus as Cause of Mastoiditis—Dr. F. P. Calhoun, Atlanta.
20. Life-saving Measures in Abdominal and Pelvic Surgery—Dr. R. R. Kime, Atlanta.
21. Subject unannounced—Dr. Monroe Smith, Atlanta.

22. Treatment of Epilepsy—Dr. E. Bates Block, Atlanta.
23. Reading of Papers Missed During Morning Session. Adjournment.

---

### REGULAR MEETING OF THE FULTON COUNTY MEDICAL SOCIETY, MAY 19, 1910.

REPORTED BY R. R. DALY, M. D.

Dr. L. Amster read a paper on "The General Principles of Hydrotherapy" which appears elsewhere in the JOURNAL.

Dr. Duncan said that he used water more to keep well than as a cure and it is successful. He has little or no experience with it as a medicine and does not know whether he could continue his baths if sick and asked Dr. Amster for information on that point.

Dr. Toepel said that he worked with Dr. Amster twelve years ago before the recent advances had been made when they were successful in their treatment by using blankets and douches. With the new experience Dr. Amster has recently gained in Vienna excellent advances have been made and these should be appreciated in Atlanta. We have neglected the natural remedies too long.

Dr. Amster said in closing that not many years ago he was indifferent to Hydrotherapy but nature treatment is getting to be better recognized and he has learned the value of the process and it is no longer the step-child of medicine. He advises every doctor to learn the fundamental principles of Hydrotherapy and its application in home treatment and to use it there. The text-books have never been complete in description of the method and the physician has to work it out for himself in the clinic. He hopes to publish a series of articles describing Hydrotherapy and its manifold applications.

Dr. Frances Bradley addressed the Society on the subject of "Preventive Medicine and Public Education" on behalf of the women physicians of the American Medical Association. She said that open public meetings had been held in several states to great advantage and she urges action of a similar character throughout Georgia. She desires that the medical profession generally endorse the plan and asks that personally they consent to give lectures on various subjects before the Mothers' Meetings and schools and that the medical society hold open public meetings on important topics for adults.

Dr. Donaldson presented a case of pus kidney which he removed yesterday. The man was injured and had some hematurea six years ago. Since then he has had greatly increasing symptoms of tumor and recently considerable fever. His preliminary operation drained about a quart of pus before the kidney was taken away. The patient is doing well this morning.

Dr. Toepel spoke at length on the matter of physical training in the schools. Several years ago he had difficulty in getting the children to take their exercise out of doors but finally accomplished it. Now the teachers ask that the indoor method be resumed. He requests that the Fulton County Medical Society enter its protest against this change.

